# Molecular mechanisms of endothelial to mesenchymal cell transition (EndoMT) in experimentally induced fibrotic diseases

**DOI:** 10.1186/1755-1536-5-S1-S7

**Published:** 2012-06-06

**Authors:** Sonsoles Piera-Velazquez, Sergio A Jimenez

**Affiliations:** 1 Jefferson Institute of Molecular Medicine, Thomas Jefferson University, Philadelphia, PA 19107, USA

## Abstract

Several recent studies have demonstrated that endothelial to mesenchymal transition (EndoMT), a newly recognized type of cellular transdifferentiation may be an important source of myofibroblasts during the development of experimentally induced pulmonary, cardiac and kidney fibrosis. EndoMT is a complex biological process induced by members of the transforming growth factor (TGF-β) family of regulatory polypeptides in which endothelial cells adopt a mesenchymal or myofibroblastic phenotype acquiring motile and contractile properties and initiating expression of mesenchymal cell products such as α smooth muscle actin (α-SMA) and type I collagen. Although these experimental studies provide compelling evidence for the participation of EndoMT in the development of experimentally-induced fibrotic processes the precise role of EndoMT in the pathogenesis of human fibrotic disorders requires confirmation and validation from studies of human clinical pathologic conditions. Such confirmation should lead to a change in the paradigm of the origin of profibrogenic myofibroblasts involved in human fibrotic diseases. Further understanding of the molecular mechanisms and the regulatory pathways involved in EndoMT may lead to the development of novel therapeutic approaches for the incurable and often devastating fibrotic disorders.

## Introduction

It is generally accepted that activated mesenchymal cells or myofibroblasts play a crucial role in the pathogenesis of various fibrotic diseases including interstitial pulmonary fibrosis, systemic sclerosis and liver or cardiac fibrosis being responsible for the exaggerated production and accumulation of extracellular matrix proteins in various organs affected by these diseases [1-4]. Although the etiologic factors that initiate the fibrotic diseases are diverse and in most instances remain unknown, the accumulation of activated myofibroblasts in affected tissues and the persistence of their elevated biosynthetic functions are crucial determinants of the severity and rate of progression of these diseases, and of their clinical course, response to therapy, prognosis, and mortality. Thus, a precise understanding of the origin of these cells and of the mechanisms involved in the regulation of their intricate functions are of paramount importance for the development of effective therapeutic approaches for the vast spectrum of disorders associated with tissue and organ fibrosis [5,6]. Myofibroblasts in the fibrotic diseases are derived from at least three sources: 1) expansion and activation of resident tissue fibroblasts [7,8]; 2) transition of epithelial cells into mesenchymal cells, a process known as epithelial-mesenchymal transition [9-14]; and 3) tissue migration of bone marrow-derived circulating fibrocytes [15,16]. Recently, endothelial to mesenchymal transition (EndoMT), a newly recognized type of cellular transdifferentiation [17], has emerged as another possible source of tissue myofibroblasts which may play a crucial role in the pathogenesis of fibrotic diseases [18,19]. EndoMT is a complex biological process in which endothelial cells lose their specific endothelial cell markers, such as vascular endothelial (VE) cadherin, and acquire a mesenchymal or myofibroblastic phenotype initiating expression of mesenchymal cell products including α-smooth muscle actin (α-SMA), vimentin, and types I and III interstitial collagens. Besides acquisition of an activated pro-fibrogenic phenotype these cells also become motile and are capable of migrating into surrounding tissues. Although in the past EndoMT was believed to be a rare phenomenon confined to certain stages of embryonic development [17,20] its occurrence in fibrotic disorders is gaining increased attention. Indeed, multiple antibody immunofluorescence confocal microscopy studies and endothelial cell lineage analyses during the development of various experimentally-induced animal models of tissue fibrosis have demonstrated the participation of EndoMT in the pathogenesis of fibrotic processes in various organs [21-26]. Although numerous studies have examined the role of epithelial mesenchymal transition (EMT) in the pathogenesis of fibrotic disorders [27] and there has been extensive investigation of the molecular events responsible for this process [28-31], studies examining the mechanisms involved in EndoMT and its potential participation in pathologic tissue fibrosis in human diseases are limited.

### EndoMT in experimentally-induced organ fibrosis

**Figure 1:**
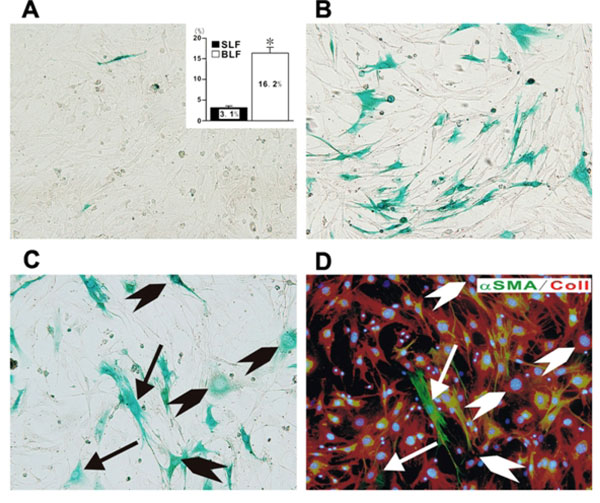
**Demonstration of endothelial cell-derived fibroblasts in fibroblast cultures established from lung parenchyma of mice with bleomycin induced pulmonary fibrosis**. Fibroblast cultures were established from lungs from mice injected intratrachealy with either normal saline or bleomycin. When the cultures reached confluency they were stained with x-gal to identify the cells from endothelial lineage. Note the absence of x-gal staining cells in the cultures from saline injected control mice (A) in contrast with the marked abundance of x-gal staining fibroblasts in the cultures from bleomycin injected mice (B). The inset in A shows the percentage of x-gal positive cells in four separate samples of cultured fibroblasts from saline injected mice (SLF) compared to eight separate samples of fibroblasts cultured from bleomycin injected mice (BLF). Figures C and D show sequential staining of a fibroblast culture from bleomycin injected mice with x-gal (C) followed by immunocytochemistry for the mesenchymal cell markers type I collagen (red) and α- SMA (green). The arrows indicate cells positive for x-gal, type I collagen, and α- SMA, whereas the arrowheads indicate cells positive for x-gal and type I collagen. Reproduced from reference 26 with permission.

The occurrence of EndoMT in experimentally induced cardiac fibrosis was originally described by Zeisberg et al. [21] employing endothelial cell lineage analysis in transgenic mice. In these studies, analyses of the proportion of fibroblasts present in the fibrotic myocardium of mice with aortic banding induced myocardial fibrosis showed that from 27 to 35% of fibroblasts originated from endothelial cells. Several other studies have confirmed the emergence of activated fibroblasts originating from endothelial cells in various experimentally induced models of cardiac fibrosis [22,23] and collectively have suggested that in these experimental conditions EndoMT represents an important contributor to the generation of fibrotic tissue and, therefore, this pathway may represent a novel therapeutic target. EndoMT has also emerged as a potentially important mechanism in the development and progression of experimentally induced pathological kidney and pulmonary fibrosis. Numerous studies have shown that EndoMT is a novel pathway leading to fibrotic development in diabetic nephropathy and other models of kidney fibrosis. An extensive study by Zeisberg et al. [24] examined the role of EndoMT in three murine models of chronic kidney disease: unilateral ureteral obstructive nephropathy, streptozotocin-induced diabetic nephropathy and a model of Alport renal disease. The results of these studies indicated that 30-50% of myofibroblasts in the fibrotic kidneys, identified by their expression of a fibroblast phenotype and α-SMA, display the endothelial cell specific CD31 surface marker indicating their endothelial cell origin. These studies were validated by endothelial cell lineage tracing and were also confirmed by studies from other laboratories [18,25]. The possible role of EndoMT in experimentally induced pulmonary fibrosis was examined by Hashimoto et al. [26]. These authors evaluated EndoMT as a source of interstitial fibroblasts in bleomycin-induced lung fibrosis using double-transgenic mice in which LacZ was stably expressed in endothelial cells and therefore allowed the histological identification of any cells originated from an endothelial cell lineage. Following endotracheal injection of bleomycin the areas of fibrotic involvement were shown to contain large numbers of fibroblasts of endothelial origin. To directly demonstrate the presence of endothelial cell-derived lung fibroblasts in affected lung fibrotic tissues, lung fibroblasts were isolated and cultured from either saline injected control mice or from mice that received bleomycin injections. These studies revealed that approximately 16% of lung fibroblasts in the cultures from bleomycin-treated mice were derived from endothelial cells as illustrated in Figure 1.

### EndoMT of human endothelial cells

In contrast with the extensive evidence from experimental animal models demonstrating the important participation of EndoMT in the development of tissue fibrosis studies with human endothelial cells are just beginning to appear. One such study [32] examined the conversion of human microvascular intestinal endothelial cells into mesenchymal cells following exposure to a combination of proinflammatory cytokines (TGF-β, TNF-α and IL-1β) *in vitro*. The treated cells displayed a progressive loss and the eventual disappearance of endothelial-specific cell markers (CD-31 and VE-cadherin) and a parallel acquisition of mesenchymal cell markers including vimentin, collagen I and N-cadherin. These results suggested that intestinal endothelial cells exposed to an inflammatory environment may participate in the intestinal fibrotic process which accompanies intestinal inflammatory diseases. The *in vitro *observations were confirmed by studies of molecular co-localization in human colonic mucosa [32].

Another study examined the expression of cell surface markers specific for endothelial cells in mesenchymal/fibroblastic cells present in the subendothelial compartment of small vessels in lungs from patients with Systemic Sclerosis-associated pulmonary fibrosis [33]. Confocal microscopy demonstrated the expression of endothelial cell markers (CD-31 and CD-34) in mesenchymal cells embedded within the subendothelial neointima of small pulmonary arteries in lung specimens from patients with Systemic Sclerosis. These two studies provide strong support to the concept that EndoMT may also play an important role in the pathogenesis of human fibrotic diseases.

### Molecular mechanisms of EndoMT

In contrast to the very extensive studies conducted to unravel the molecular mechanisms and the regulatory pathways that modulate epithelial mesenchymal transition [27-31], the mechanisms involved in the EndoMT process have not been explored in detail. Few studies have examined the biochemical and molecular changes occurring in endothelial cells during their transdifferentiation into mesenchymal myofibroblasts and the regulatory events involved in this process. It is generally accepted that TGF-β plays a crucial role in tissue fibrosis and is implicated in the pathogenesis of numerous fibrotic disorders [5,6,34-37]. Recent studies have shown that besides causing a potent stimulation of the expression of numerous genes participating in the fibrotic process, TGF-β is involved in the generation of myofibroblasts through EndoMT [21,22,38-42]. The studies in experimentally induced cardiac hypertrophy provided an elegant demonstration of the role of TGF-β in this process [21]. These studies showed that TGF-β was a crucial mediator causing endothelial cells to undergo EndoMT since there was a significant reduction in the number of mesenchymal cells originating from endothelial cells when the TGF-β response was blunted by the deficiency of Smad3 in Smad 3+/- transgenic mice. These results have been confirmed employing a TGF-β receptor kinase inhibitor which inhibits activated TGF-β [40] as well as several small molecule inhibitors of intracellular phosphorylation reactions [41,42].

Besides TGF-β it has been shown that endothelin-1 (ET-1) may also participate in EndoMT. In one study, Widyantoro et al. [23] showed that endothelial cell-derived ET-1 promotes cardiac fibrosis and heart failure in diabetic hearts through stimulation of EndoMT. These features were abolished in hearts from transgenic mice with endothelial cell specific ET-1 deletion. In another study it was shown that in vitro treatment of murine pulmonary endothelial cells with ET-1 although not capable of initiating EndoMT by itself could excert a powerful synergistic effect with TGF-β (Li and Jimenez; unpublished observations).

Although there are numerous studies which have conclusively shown the crucial involvement of members of the TGF-β family of growth factors in initiation of EndoMT, the detailed molecular events and the intracellular cascades activated by TGF-β that result in the remarkable phenotypic change of endothelial cells to mesenchymal cells have not been entirely elucidated. Recent studies in primary cultures of murine pulmonary endothelial cells [41] and in cultured human dermal microvascular cells [42] demonstrated that EndoMT involves both Smad-dependent and Smad-independent pathways. The downstream signaling pathway initiated by TGF-β resulted in strong upregulation of the transcriptional repressor Snail1. Snail1 causes potent inhibition of E-cadherin gene transcription in cultured cells and plays an important role in the epithelial to mesenchymal transition [27,28] indicating that the EndoMT process shares similar molecular mechanisms with epithelial mesenchymal transdifferentiation.

**Figure 2:**
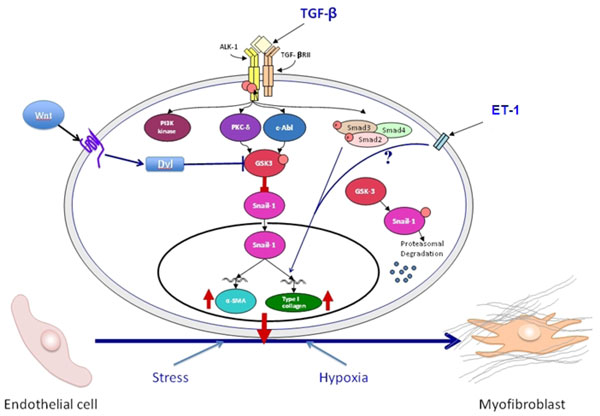
**Schematic diagram showing the putative TGF-β signaling pathways involved in EndoMT**. The diagram shows the numerous putative pathways that may participate in the EndoMT process and may be involved in the pathogenesis of human fibrotic disorders. One central pathway initiated following ligand-binding activation of the Smad-independent TGF-β pathway causes phosphorylation of GSK-3β mediated by PKC-δ and the cAbl non-receptor kinase. Phosphorylation of GSK-3β at serine 9 (ser9) causes its inhibition which then allows Snail1 to enter the nucleus. Nuclear accumulation of Snail1 results in marked stimulation of Snail1 expression which then leads to acquisition of the myofibroblast phenotype with stimulation of α-SMA. The inhibition of GSK-3β ser9 phosphorylation by specific inhibition of PKC-δ or c-Abl activity allows GSK-3β to phosphorylate Snail1 targeting it for proteosomal degradation and thus, effectively abolishes the acquisition of the myofibroblastic phenotype and the fibrotic response. Other pathways such as the ET-1, Wnt, hypoxia and cellular stress pathways may also participate although the molecular events have not been fully elucidated. Modified from Li and Jimenez [37].

Several studies explored the underlying molecular pathways that may cause the significant loss of endothelial-specific markers while inducing strong *de novo *mesenchymal phenotypes. These studies identified the c-Abl protein kinase (c-Abl), protein kinase Cδ (PKC-δ), and glycogen synthase kinase 3β (GSK-3β) as important participants and that GSK-3β kinase phosphorylation was a crucial event in this process [41,42]. It is well known that phosphorylation of specific serine residues in GSK-3β results in inactivation of the kinase which in turn induces the nuclear accumulation of Snail1 followed by a profound increase in the expression of its corresponding gene. The transcriptional effects of Snail1 induce the expression of a mesenchymal cell-specific phenotype although the precise mechanisms involved remain obscure. In contrast, in the absence of GSK-3β phosphorylation the GSK-3β kinase is active and induces the proteosomal degradation of Snail1, thus abrogating the acquisition by endothelial cells of a mesenchymal cell phenotype. Other studies have shown that several important regulatory pathways including the canonical Wnt pathway, the HIF-1α hypoxia induced pathway, and the response to cellular stress may also participate in the regulation of EndoMT [43,44] as illustrated in Figure 2.

In summary, numerous recent studies have provided strong evidence for the prominent participation of EndoMT in the generation of activated myofibroblasts during the development of experimentally induced tissue fibrosis suggesting that EndoMT plays a crucial role in fibrotic diseases. There is some early experimental evidence providing support and validation for the participation of EndoMT in the development of human intestinal fibrosis and of Systemic Sclerosis-associated pulmonary fibrosis. The firm demonstration of the occurrence of EndoMT in human fibrotic diseases and further understanding of the molecular mechanisms involved may lead to the pharmacologic modulation or abrogation of this pathway in human fibrotic disorders and may represent a novel therapeutic approach for these devastating diseases.

## Competing interests

The authors declare that they have no competing interests.
